# 775 Increased Reimbursement by Decreasing the Mortality Index Using GLIM in the Elderly for Malnutrition

**DOI:** 10.1093/jbcr/irad045.250

**Published:** 2023-05-15

**Authors:** Jamie Hollowell, Aryn Cruz, Molly Marsh, Booker King, Felicia Williams, Eli Maxwell

**Affiliations:** UNC Jaycee Burn Center, Chapel Hill, North Carolina; UNC Jaycee Burn Center, Chapel Hill, North Carolina; UNC Jaycee Burn Center, Chapel Hill, North Carolina; UNC Jaycee Burn Center, Chapel Hill, North Carolina; University of North Carolina at Chapel Hill, Chapel Hill, North Carolina; UNC Jaycee Burn Center, Chapel Hill, North Carolina; UNC Jaycee Burn Center, Chapel Hill, North Carolina; UNC Jaycee Burn Center, Chapel Hill, North Carolina; UNC Jaycee Burn Center, Chapel Hill, North Carolina; UNC Jaycee Burn Center, Chapel Hill, North Carolina; University of North Carolina at Chapel Hill, Chapel Hill, North Carolina; UNC Jaycee Burn Center, Chapel Hill, North Carolina; UNC Jaycee Burn Center, Chapel Hill, North Carolina; UNC Jaycee Burn Center, Chapel Hill, North Carolina; UNC Jaycee Burn Center, Chapel Hill, North Carolina; UNC Jaycee Burn Center, Chapel Hill, North Carolina; University of North Carolina at Chapel Hill, Chapel Hill, North Carolina; UNC Jaycee Burn Center, Chapel Hill, North Carolina; UNC Jaycee Burn Center, Chapel Hill, North Carolina; UNC Jaycee Burn Center, Chapel Hill, North Carolina; UNC Jaycee Burn Center, Chapel Hill, North Carolina; UNC Jaycee Burn Center, Chapel Hill, North Carolina; University of North Carolina at Chapel Hill, Chapel Hill, North Carolina; UNC Jaycee Burn Center, Chapel Hill, North Carolina; UNC Jaycee Burn Center, Chapel Hill, North Carolina; UNC Jaycee Burn Center, Chapel Hill, North Carolina; UNC Jaycee Burn Center, Chapel Hill, North Carolina; UNC Jaycee Burn Center, Chapel Hill, North Carolina; University of North Carolina at Chapel Hill, Chapel Hill, North Carolina; UNC Jaycee Burn Center, Chapel Hill, North Carolina; UNC Jaycee Burn Center, Chapel Hill, North Carolina; UNC Jaycee Burn Center, Chapel Hill, North Carolina; UNC Jaycee Burn Center, Chapel Hill, North Carolina; UNC Jaycee Burn Center, Chapel Hill, North Carolina; University of North Carolina at Chapel Hill, Chapel Hill, North Carolina; UNC Jaycee Burn Center, Chapel Hill, North Carolina

## Abstract

**Introduction:**

One of the primary factors for burn wound healing is adequate nutrition. Many burn patients require supplemental nutrition during their burn care, however, their pre-existing nutritional status is also a factor in their morbidity and mortality. Malnutrition on admission or during their hospital stay has a significant impact on outcomes including wound healing, infection, and length of stay, along with effects on reimbursement and mortality outcomes predictors. American Society for Parenteral and Enteral Nutrition (ASPEN) criteria have long been used to assess malnutrition in burn patients in our institution though this may not be the ideal assessment tool, especially in patients over 65 years of age. In January 2016, the Global Leadership Initiative on Malnutrition (GLIM) working group created an updated assessment for malnutrition that takes additional factors into account. We aimed to compare ASPEN to GLIM criteria to determine if there is a difference in the number of patients meeting the criteria for malnutrition in the elderly.

**Methods:**

All patients admitted between July 2013 to June 2021, 65 years or older, burn or skin disorder of at least 20% total body surface area, designated as Normal or underweight based upon body mass index, with registered dietitian assessments and lengths of stay of at least 2 days were included. Patients were identified using the Institutional Burn Center registry, and linked to the clinical and administrative data. Primary outcome was whether or not those patients triggered malnutrition diagnosis based upon ASPEN or GLIM criteria.

**Results:**

Three hundred sixty-five patients were included in the study. Twenty-five patients had burns over 20%, and 14 met the criteria for nutritional assessment. Using ASPEN criteria, 2 out of 14 patients met criteria for malnutrition. Of those same patients, 8 out of 14 patients met criteria for malnutrition using GLIM assessment.

**Conclusions:**

While this study is not powered for statistical analysis, the results demonstrate that GLIM may be a valuable adjunct to ASPEN to assess malnutrition in burn patients, especially in those patients over 65 years of age. Further study is needed to examine whether GLIM would be a superior tool or at least a worthy addition to our nutritional analysis. Burn patients have a unique need for adequate nutrition to support healing, and accurate evaluation of their requirements warrants a precise tool.

**Applicability of Research to Practice:**

This study may demonstrate the need for additional evaluation of GLIM as a replacement or adjunct tool for malnutrition in the burn population. This study may also suggest the need for further assessment of other tools to evaluate nutrition on in this population. This study may also suggest impact on mortality index and length of stay that affect reimbursement and rankings.

